# Potential drug–drug interactions in HIV-perinatally infected adolescents on antiretroviral therapy in Buenos Aires, Argentina

**DOI:** 10.7448/IAS.17.4.19764

**Published:** 2014-11-02

**Authors:** Ezequiel Cordova, Diego Cecchini, Claudia Rodriguez

**Affiliations:** Infectious Diseases Unit, Hospital Cosme Argerich, Buenos Aires, Argentina

## Abstract

**Introduction:**

An increasing number of treatment-experienced perinatally HIV-infected adolescents (PHA) are being transitioned from paediatric centres to adult HIV-care [1]. Most of them had been heavily exposed to antiretroviral drugs (ARVs), harbour drug-resistant viruses and require non-antiretroviral medication due to comorbidities [2]. This may predispose for clinically significant drug–drug interactions (CSDDIs) [3]. There are no studies concerning CSDDIs in PHA. We aimed to evaluate the prevalence of concomitant medications and CSDDIs in PHA who were transitioned for adult HIV-care to the Infectious Diseases Unit, Cosme Argerich Hospital, Buenos Aires City, Argentina.

**Materials and Methods:**

Descriptive pilot cross-sectional study (March to June 2014). PHA under ARVs at the time of the study were assessed for concomitant medication. CSDDIs were screened and categorized using the University of Liverpool Drug Interactions Program (www.hiv-druginteractions.org) [4].

**Results:**

Forty-five patients were included. Female sex: 53%. Median (IQR) age: 20 years (18–22). CDC-stage C was observed in 27 (79%); 50% had ≥1 comorbidities including 3 with HCV co-infection. Drug abuse was observed in 6 (13%). The median of prior ARV regimens was 3 (3–5). Current ARV regimen included: PI: 87%, NNRTI: 27%, INSTI: 20%, enfuvirtide: 7% and CCR5 inhibitor: 4%. Median CD4 T-cell count: 568 cells/mL (279–771). Viral load <50 copies/mL: 80%. Sixty percent (27/45) had ≥1 co-medications (median 1). The most frequent co-medications were NSAIDs (40%), hormonal therapy (19%) and antimicrobials (19%). Use of herbal supplements was observed in 10 (22%). Overall, 23 (51%) had ≥ 1 CSDDIs: 19/27 (70%) with co-medication (orange flag=18 and red flag=1); and 2/10 (20%) with herbal supplements. ARV–ARV interactions were observed in 4/45 (9%): unboosted atazanavir+tenofovir (n=2), unboosted atazanavir+efavirenz (n=1) and lopinavir/ritonavir+efavirenz (n=1) (all orange flag). Considering patients with CSDDIs, 6 (26%) had a CSDDI that could reduce ARV levels.

**Conclusions:**

In this pilot study, a high prevalence of comorbidities, co-medications and CSDDIs was observed in PHA. A considerable proportion of patients had CSDDIs with a potential to cause sub-therapeutic ARV levels, what could be a concern in patients harbouring drug-resistance viruses. Therefore, clinicians should be aware of comorbid conditions pharmacologic management in order to avoid CSDDIs with ARVs agents.
